# Replacement of Sodium Tripolyphosphate with Iota Carrageenan in the Formulation of Restructured Ostrich Ham ^†^

**DOI:** 10.3390/foods10030535

**Published:** 2021-03-05

**Authors:** Sumari Schutte, Jeannine Marais, Magdalena Muller, Louwrens C. Hoffman

**Affiliations:** 1 Department of Animal Sciences, University of Stellenbosch, Private Bag X1, Matieland, Stellenbosch 7602, South Africa; sumari.milner@gmail.com; 2 Department of Food Science, University of Stellenbosch, Private Bag X1, Matieland, Stellenbosch 7602, South Africa; jeanninemarais@sun.ac.za (J.M.); mm7@sun.ac.za (M.M.); 3 Centre for Nutrition and Food Sciences, Queensland Alliance for Agriculture and Food Innovation (QAAFI), The University of Queensland, Health and Food Sciences Precinct, 39 Kessels Rd., Coopers Plains 4108, Australia

**Keywords:** iota carrageenan, chemical composition, consumer acceptance, descriptive analysis, ham, ostrich, phosphate, polysaccharide, processed, restructured meat, sensory profile

## Abstract

The influence of iota carrageenan (iota-CGN) as a partial replacement of sodium tripolyphosphate (STPP) was investigated on the physical (pH, yield, instrumental color, texture profile analysis), chemical (moisture, protein, total fat, ash, phosphate) and sensory (descriptive analysis, acceptance testing) quality of restructured ostrich ham (95% lean meat plus fat). Treatments consisted of five decreasing levels of STPP (0.70%, 0.53%, 0.35%, 0.18% and 0%) that were simultaneously substituted with five increasing levels of iota-CGN (0%, 0.1%, 0.2%, 0.3% and 0.4%). Cooked yield, hardness, cohesiveness, and gumminess of restructured ostrich ham increased (*p* ≤ 0.05) with decreasing levels of STPP (and increased levels of iota-CGN). No significant trend in instrumental color measurements or springiness were observed between treatments. Ostrich ham with 0.35% STPP and lower had increased ostrich meat aroma and flavor, while spicy aroma and flavor, mealiness and consumer acceptance decreased. Iota carrageenan can be substituted for STPP (up to 0.35% STPP and 0.2% iota-CGN) to produce reduced STPP ham.

## 1. Introduction

Restructured ham is usually prepared from large pieces of meat that are molded together to resemble a whole muscle meat product after cooking. The actual binding of adjacent meat pieces relies on extraction of myofibrillar proteins by salt (NaCl), phosphate and mechanical action (massaging or tumbling). During subsequent heating, the latter proteins, of which myosin is the major protein, coagulate and act as a bonding agent holding the meat pieces together [1,2,3,4,5]. The binding properties of restructured ham are essential to produce a uniformly attractive product with desirable slicing characteristics. The most desirable properties of high-quality cooked ham are cohesiveness, textural firmness, and juiciness.

Polyphosphates are used extensively in restructured meat products due to their functional properties of increasing binding strength, water holding capacity and yield [4,6,7,8,9,10]. Polyphosphate action is ascribed to the increase in the pH and ionic strength in meat products [11,12]. Tripolyphosphates (TPP) are the most widely used of all the phosphates utilized in meat processing and are typically permitted up to 3.5% of final product weight. However, there is an increase in the demand for meat products with reduced phosphate levels [13].

The presence of excessive amounts of phosphates in the diet may influence the calcium, iron, and magnesium balance in the human body, and can increase the risk of bone diseases [14,15,16]. Furthermore, consumers and retailers generally associate polyphosphates with cost reduction and lower quality products. Consumers also seem to associate the term ‘polyphosphates’ with non-food applications, viewing them as ‘chemical products’. The former indicates an opportunity for the use of alternatives to phosphates in restructured cooked meat products [3,5,13,17]. Numerous non-meat functional ingredients, mainly proteins and polysaccharides, have been applied as binders, fillers, and extenders to improve the quality of restructured meat products [4,5,18,19]. These ingredients are primarily used for their water binding ability and texture modification functionality [20].

Hydrocolloids with their unique characteristics in building texture, stability and emulsification are of great interest in the low-fat processed meat area due to their ability to bind water and form gels [21]. Carrageenan (CGN), a sulphated polysaccharide extracted from seaweed, is a hydrocolloid used extensively in the food industry in a broad range of applications because of its water binding, thickening and gelling properties [22,23]. There are three major types: kappa (κ, gelling); iota (ι, gelling); and lambda-CGN (λ, non-gelling). These differ in degree and manner of sulphation, the position of the 3–6 anhydrogalactose residues, their pyranose ring conformations, and the cations associated with the sulphate groups [23]. Carrageenans, alone or combined with other ingredients, have been used extensively in restructured meat products [24,25,26,27,28,29] for their ability to form gels, retain water and to provide a desirable texture [30,31]. Various levels of ingredients in combination with CGN have been studied; for example, the use of 1.5% salt with iota-CGN improved the cooking yield, juiciness, and tenderness of restructured pork nuggets [25]. Kappa-CGN favorably affected hydration properties and thermal stability, yielding lower cooking loss, purge, and expressible moisture of beef gels [27], whilst kappa-CGN increased the sliceability and rigidity in roasted turkey breasts [24], and improved the adhesion in pork hams [26].

Ostrich meat is frequently marketed as a healthy alternative to other red meats as it has a favorable fatty acid profile and a low intramuscular fat content [32,33,34]. Ostrich meat has a high ultimate pH of ca. 6.0 [35] and should by implication have a high-water binding capacity and thus be able to retain high levels of moisture. Therefore, moisture-retaining agents, such as phosphates, in restructured ostrich meat products could be reduced.

To maintain the health characteristics of ostrich meat, it is suggested that an alternative ingredient, that mimics the textural, functional and flavor characteristics of phosphate, be introduced in the formulation of restructured meat products. Therefore, the aim of this study was to investigate the effect of replacement of sodium tripolyphosphate (STPP) with iota carrageenan (iota-CGN) on the physical, chemical, sensory characteristics, and consumer acceptability of restructured cooked ostrich ham.

## 2. Materials and Methods

### 2.1. Ham Manufacture

Five different ham formulations with decreased levels of STPP and increased levels of iota-CGN were produced (Table 1). Each treatment was formulated to contain a 95% Total Meat Equivalent (TME) on chemical analysis (lean meat and fat). Brine ingredients, expressed as percentage in the brine, consisted of 9% NaCl, 0.25% sodium erythorbate, 1% curing salt (NaCl + 0.6% nitrite), 20% starch (corn flour), 1% ground garlic, 1% ground ginger, STPP (3.5%, 2.63%, 1.75%, 0.88% and 0%, respectively), iota-CGN (0%, 0.5%, 1.0%, 1.5% and 2.0%, respectively), and water (64.25%, 64.62%, 65.00%, 65.37% and 65.75%, respectively). The corn flour was added to the brine and the meat after the first tumble cycle.

Ostrich (*Struthio camelus* var. *domesticus*) fan fillets (n = 40 different birds; 1–1.5 kg weight per fan fillet) were obtained from a local European Union approved abattoir, Mosstrich (Mossdustria, Mossel Bay, South Africa), with all the muscles being randomly sampled from one day’s kill. The meat was vacuum packed and frozen before being transported to Stellenbosch; where it was stored at −20 °C until used. Iota-CGN (GENU^®^ texturizer type MB-150F) from Tranarc (Tranarc Holdings Pty Ltd., Benmore, South Africa) was used. All the remaining ingredients were provided by a single provider, Deli Spices (Epping, Cape Town, South Africa).

The thawed (24 h at 4 °C) ostrich fan fillets (n = 10 fillets per batch) were cut into fist sized pieces (±100 g per piece) and mixed in a container. The meat structure was subsequently further disrupted by the mild shearing action of passing through a meat mincing machine without any cutting blades or plates. The latter opened the meat structure to facilitate brine penetration and protein extraction, without reducing particle size. The meat from each batch was then divided into five smaller batches—one batch per treatment. The brine mixture for each treatment was then added to the meat and the latter mixture was tumbled (Biro VTS-41) under vacuum (25 kPa) for 6 h (4 °C) with a cycle of 20 min tumble and 10 min rest. After tumbling, the ham mixtures were vacuum stuffed (Talsa Model T0101, Germany) into impermeable plastic casings. The above-mentioned procedures were followed four times to produce four replications per treatment. Each replicate sample weighed approx. 1.5 kg and was 30 cm in length and 12 cm in diameter. Each stuffed casing within each treatment was weighed and cooked in a water bath until a core temperature of 72 °C was reached (approximately 1 h). The internal temperature of the ham was measured using a thermocouple probe inserted into the center of the product. After cooking, the hams were immediately immersed in cold water containing ice for 15 min before refrigeration at 4 °C prior to subsequent analyses.

### 2.2. Chemical Analyses

Homogenized samples of the five ham treatments (of a randomly selected ham within each treatment) were analyzed in duplicate for total percentages of moisture, ash, and phosphorus (according to AOAC Official Methods 934.01, 942.05, and 960.03, respectively) [36]. The total crude protein content was determined on dried (60 °C for 24 h), defatted and ground (with a pestle and mortar to a fine powder) samples (0.1 mg) encapsulated in LecoTM foil sheets and analyzed using a Leco Protein Analyzer (FP-528, Leco Corporation). An EDTA calibration sample (Leco Corporation, St. Joseph, HI, USA, Part number 502–092, lot number 1038) was analyzed before and after every 10 samples, with the intention of ensuring the accuracy and recovery rate of each sample. A Nitrogen conversion factor of 6.25 was used to determine the total protein content. The total fat content was determined by extracting the fat with a 2:1 mixture of chloroform:methanol [37]. The laboratory at the Department of Animal Sciences, Stellenbosch University, is accredited by the Agricultural Laboratory Association of South Africa (AgriLASA) to perform accurate and reliable proximate analyses. For validation of accuracy and repeatability, the laboratory partakes in the monthly National Inter-laboratory Scheme where blind tests are conducted. The lean meat equivalent (LME) was calculated using a conversion factor of 30 to convert protein to lean meat and the total meat equivalent (TME) was obtained through the summation of the LME and fat.

### 2.3. Physical Analyses

The pH of the refrigerated (4 °C) cooked hams was measured with the use of a calibrated (standard buffers pH 4.0 and 7.0) portable Testo 502 pH-meter. Cooked yield, color (CIE lightness L*, a* and b* color coordinates) and Texture Profile Analysis (TPA) measurements were recorded on each of the four ham replicates per treatment. Cooking yield was expressed as follows:

Cooked yield (%) = (W1 − W2) × 100 where W1 = ham weight after cooking and W2 = ham weight before cooking

The weight of the cooked product was recorded after 24 h chilling (4 °C), when the products were removed from the casings, touch dried with absorbent paper, and casing weight recorded, separate from product weight. Product weight losses occurred primarily during thermal processing; weight loss due to the exudate remaining in the tumbler was small (about 1%) as the tumbler surfaces had been scraped with a spatula to reclaim as much exudate as possible.

Instrumental color measurements of cooked ham were recorded on three slices obtained from each of the four ham replicates per treatment [38]. A color-guide 45°/O° colorimeter (Cat no: 6805; BYK-Gardner, BYK-Instruments, Orlando, FL, USA) was used; the colorimeter was calibrated using the supplied calibration white tile according to the supplier’s instruction before and between every 10 samples. Three ham slices (1.5 to 2.0 cm thick) of each treatment were allowed to “bloom” for 30 min at ambient temperature (*ca.* 20 °C) prior to color measurements. Four color measurements were recorded for each slice at randomly selected positions and expressed by the coordinated L*, a* and b* of the CIELab colorimetric space. In the color space L* indicates lightness and a* and b* are the chromaticity coordinates, where a* is the red-green range, and b* the yellow-blue range of the color spectrum.

Instrumental textural properties were analyzed using the Instron Universal Testing Machine (UTM, model 3344, 825 University Ave, Norwood, MA, 02062–2643, USA). Texture Profile Analysis (TPA) was performed on five cores (2.5 cm height and 2 cm diameter) per slice (two slices of each of the four replicates within the five treatments = 40 measurements per treatment). The cores were placed on the platform of the UTM. A circular plate of 2.5 cm diameter was attached to a 50 N load cell and the sample was compressed to 50% of its original height at a cross head speed of 200 mm/min twice in two cycles [39]. Hardness (N), springiness (mm), cohesiveness (ratio) and gumminess (N) were calculated for each sample [39].

### 2.4. Sensory Evaluation

#### 2.4.1. Descriptive Sensory Analysis

Descriptive sensory analysis (DSA) was conducted to determine the effect of STPP reduction on the sensory quality characteristics of all five treatments of ostrich ham [40]. For each treatment four replicate encased hams were produced. The encased hams (stored at 4 °C) were opened 2 h prior to sensory analysis, sliced into 3.5 mm thick slices and vacuum packed (Multivac C200, Bahnhofstraße 4, D-87787 Wolfertschwenden, Germany). Four slices were placed next to each other and the slices did not overlap when vacuum packed.

A panel of assessors (n = 8), with extensive experience in DSA of meat, was trained in two interactive sessions to familiarize them with the treatments and to identify the aroma, flavor and mouthfeel characteristics associated with the respective treatments. Reference standards were also used to enable the assessors to calibrate their sensory perception during training, thereby allowing them to recognize and score all the characteristics tested in the respective treatments. The reference standards included commercial beef fillet, ostrich fan fillet and pork ham, resembling the meaty, ostrich meat and spicy aroma and flavor notes, respectively. Beef liver was used to illustrate a mealy meat texture. The questionnaire was compiled during the first training session and refined and tested during the second training session. Unstructured 100-point line scales were used to analyze the sensory characteristics. Table 2 depicts the sensory characteristics and definitions used.

Sensory testing was performed in individual booths fitted with Compusense^®^ software (Compusense, Guelph, ON, Canada) in a temperature—(20 °C) and light-controlled (equivalent to daylight) sensory evaluation area. A sample of each of the five treatments was served to the assessors in a randomized order in four replicate test sessions (two sessions per day). The sample size per treatment per test session was one slice, with each assessor receiving an eighth (⅛) of a slice. Each sample was coded with a three-digit blinding code and served at a refrigeration temperature of ca. 6–10 °C. Assessors were provided with distilled water, dried apple pieces and water biscuits as palate cleansers.

#### 2.4.2. Acceptance Testing

Sensory acceptance testing was conducted with a hundred target consumers (79 females, 21 males) recruited among staff and students at Stellenbosch University, Stellenbosch, South Africa. The consumers tested three of the treatments (STPP levels 0.70%, 0.35% and 0.00%), without any knowledge of the formulation of the products. The sample size per treatment per consumer was an eighth (⅛) of a slice. Samples were coded with three-digit blinding codes and served in a random order to each consumer at a refrigeration temperature of ca. 6–10 °C. Testing was done in a temperature- (20 °C) and light-controlled (equivalent to daylight) sensory evaluation area. Consumer acceptance testing was tested using the traditional nine-point hedonic scale ranging from 1 (dislike extremely) to 9 (like extremely) [40].

### 2.5. Statistical Analysis

The experimental design consisted of five treatments and four replicates per treatment. One-way analysis of variance (ANOVA) was performed to compare treatment means in terms of chemical, physical and sensory data, using SAS version 9.1 statistical software [41]. The Shapiro–Wilk test was performed to test for non-normality [42]. In some cases, deviations from normality were the cause of one or two outliners, which were removed before the final analysis [43]. Student’s t-Least Significant Difference (LSD) was calculated at a 5% significant level to compare treatment means. Pearson correlation coefficients were also calculated to measure the strength and direction of the linear relationship between selected variables.

For the consumer data, hedonic score values of three of the treatments were subjected to one-way ANOVA. Student’s t-Least Significant Difference (LSD) was calculated at a 5% significant level to compare treatment means.

## 3. Results and Discussion

### 3.1. Chemical and Physical Characteristics

The chemical composition, total meat equivalent (TME), product pH, cooking yield, textural properties, and instrumental color of the five ham treatments with decreasing levels of STPP are presented in Table 3.

#### 3.1.1. Chemical Composition

The ham formulated with 0.18% STPP presented the highest moisture content of 74.3% that differed (*p* ≤ 0.05) from the hams formulated with 0.70%, 0.53% and 0% STPP (Table 3). As expected, since no fat was added during the manufacturing process, there were no differences (*p* > 0.05) in the lipid and protein content between the five ham treatments. In an earlier study [44], the lipid content of restructured pork shoulder was found to be in a range of 23% to 25%. This is much higher than the lipid content (2.5% to 2.9%) in the present study, which could be attributed to the low intramuscular fat content of ostrich meat [32]. The ash content decreased (*p* ≤ 0.05) with decreased levels of STPP; the ham formulated with 0.70% STPP had the highest ash content (4.0%) whilst the ham formulated with 0% STPP had the lowest (3.2%). As the spice content was kept constant, the decrease in ash content may be attributed to the decreasing STPP levels. As expected, the phosphorus content in the hams also decreased with decreasing levels of STPP. However, the phosphorus content measured in the end-product proved to be much higher than the expected calculated phosphate content. These elevated values could be due to the natural phosphorus content (0.51%) of the meat as reflected in the ham formulated with no STPP added to the brine. Since a constant amount of phosphate was incrementally decreased in the formulation, it could be assumed that the discrepancies in the elevated phosphorus values were due to either sampling error or increased phosphorus content of a specific batch. Decreasing levels of STPP were found to have no effect on the pH of the cooked product.

#### 3.1.2. Total Meat Equivalent (TME)

In this study the TME values of the hams formulated with 0.70%, 0.53% and 0% STPP were higher than the targeted value of 95% and therefore exceeded legal requirements, whereas the TME value of the 0.18% STPP level ham was lower (93.28%) (Table 3). Once more, the reason for this variation is unknown but may be linked to the latter sample having a lower protein and higher (*p* ≤ 0.05) moisture content thus resulting in the calculated difference.

#### 3.1.3. Cooked Yield

The decrease in STPP levels with a concomitant increase in iota-CGN levels resulted in an increase (*p* ≤ 0.05) in the cooked yield of the restructured ostrich ham (Table 3). The latter can be attributed to the gelling properties and increased water binding capacity of the increased iota-CGN content [4]. During cooking, water and water-soluble components are released from myofibrils caused by the heat denaturation of the muscle proteins. Carrageenan develops a gel layer on the surface of the ham, which has a sealing effect, thereby decreasing the loss of the internal components [4]. The cooked yield levels observed in this experiment (86.0% to 94.1%) are substantially lower than that reported by Fisher and co-workers [44], who found that an ostrich ham-like product formulated with 0.3% and 1.5% phosphate produced a cooking yield of 99.21% and 99.42%, respectively. This difference could be due to different processing techniques, i.e., Fisher and co-workers [44] tumbled the meat for 20 min, whereas in this study, the meat was tumbled for 6 h.

#### 3.1.4. Instrumental Color

The lightness (L* value) of the samples ranged between 48.1 and 51.7, redness (a* value) between 8.3 and 9.8 and yellowness (b* values) between 11.4 and 13.0 units (Table 3). The ham formulated with 0.35% STPP, was found to be the lightest (51.7) and least red (8.3) in color. However, the instrumental color measurements of the different ostrich ham samples revealed no pattern with relation to the decrease in STPP levels. This result is supported by a visually observed variation in the composition of each of the sample slices. Ostrich meat is known to have a darker color than other red meat types [45]. This is also evident in this study where the range of a* values (redness) in ostrich ham (8.3 to 9.8) are much higher than that of, for example, restructured beef steaks (3.82 to 5.94) [46]. Though not measured, it was observed that storage of the chilled (<4 °C) ham under lighting conditions (exposure of ham to light) between manufacture and consumption (over a 2-week period) led to browning of the product (decrease in redness). Light has a pro-oxidant effect that provokes a decrease in a* values due to oxidation and degradation of the nitroso-pigment [47,48]. This rapid oxidation warrants further investigation as does the use of higher nitrite levels to minimize this phenomenon.

#### 3.1.5. Instrumental Texture Properties

The effect of the variation of the composition within each sample slice was reflected in the results for instrumental texture as no significant pattern was observed with the incremental decrease in the STPP levels (Table 3). However, significant differences in hardness, cohesiveness and gumminess were only observed with relation to the extreme manipulation of STPP (0.70% and 0%) during this experiment. The 0.53%, 0.35% and 0.18% STPP levels did not have a significant effect on the mentioned characteristics. The observed increase in the measured textural properties may be the results of increased levels of iota-CGN that forms a firm cohesive gel structure during cooling. These findings agree with results by Ulu [49], who studied the effect of carrageenan on the cooking and textural properties of low-fat meatballs.

### 3.2. Sensory Characteristics and Consumer Acceptance

The effect of reduced STPP on the sensory profile of five ham treatments is shown in Table 4. A meaty aroma was found to be the highest in the ham formulated with 0.35% (30.9), followed by 0.18% and 0.70% (25.7 and 25.4, respectively) STPP. Additionally, the ham formulated with 0.35% STPP was found to have the strongest (*p* ≤ 0.05) meaty flavor, compared to the other ham treatments. All ham treatments illustrated perceptible meaty aromas and flavors, irrespective of STPP level. Ostrich meat aroma and flavor for the ham formulated with 0.18% and 0% STPP was found to be much stronger (*p* ≤ 0.05) than the other ham treatments. The assessors were not able to discriminate (*p* > 0.05) between the ham treatments formulated with 0.70%, 0.53% and 0.35% STPP in terms of ostrich meat aroma and flavor. Therefore, a STPP level in ostrich ham of 0.18% and lower, does not conceal the typical aroma and flavor of ostrich meat even though spices were included at a constant level in all five treatments. Ginger and garlic were included in the formulae to mask the typical ostrich meat aroma and flavor. The sensory assessors perceived a slight spicy aroma and flavor in all ham treatments, which was perceived at lower intensities in the ham treatments with lower STPP levels (0.18% and 0%). Mealiness was defined as the mouthfeel experienced when the meat pieces separate upon chewing. This perception is indicative of the degree of cohesion between the meat pieces of the restructured ham. It seemed that STPP levels of 0.35% and higher resulted in increased mealiness (*p* ≤ 0.05), significantly more than STPP levels 0.18% and 0.00%. Mealiness also correlated negatively (*r* > −0.9; *p* ≤ 0.05) with the instrumental textural properties, particularly with the instrumental variables, hardness, and cohesiveness [49,50]. This increased mealiness could also be attributed to the increased cooking loss (Table 3) experienced in the higher % STPP inclusion treatments. This indicates that decreasing levels of STPP (coupled with increasing levels of iota-CGN) has a negative impact on the textural quality of the product as perceived by a trained taste panel.

Table 4 illustrates the degree of liking, as perceived by a group of target consumers, for three of the ostrich ham treatments. This group of consumers equally liked the ham formulated with 0.70% and 0.35% STPP (*p* > 0.05). However, the ostrich ham prepared with 0% STPP was found to be significantly (*p* ≤ 0.05) less liked (an average value of 5.4 translates to neither like nor dislike on the nine-point hedonic scale). Therefore, it can be concluded that the STPP level in ostrich ham can be successfully reduced to an acceptable level of 0.35%. These results serve as a further confirmation that further product development is necessary to produce a feasible phosphate-free ostrich ham to the consumer [51].

## 4. Conclusions

The results from this study indicate that the production of a reduced STPP ostrich ham is a viable option for the ostrich meat industry. Due to the variation in the composition within the replicate samples of each treatment, no significant tendency was found with decreasing levels of STPP with relation to the chemical composition and physical properties measured. However, decreasing levels of STPP showed significant increases in the cooked yield, which could be attributed to the water binding ability of the increased levels of iota-CGN. The low-fat content of ostrich ham makes it a healthy option for the consumer. Descriptive sensory analysis and consumer acceptance results revealed that the STPP level in ostrich ham could be reduced to an acceptable level of 0.35%. Further research should investigate the use of other alternatives to substitute phosphate compounds and focus on optimizing the processing technique (i.e., tumbling time) for optimum myofibrillar protein extraction to manufacture a product with optimum textural and sensory quality. Further research should also include the use of antioxidants to control color changes and shelf-life studies of the product.

## Figures and Tables

**Table 1:** Formulation of five ostrich ham treatments.

	Sodium Tripolyphosphate/Iota Carrageenan Levels				
Ingredients (%)	0.70%/0.0%	0.53%/0.1%	0.35%/0.2%	0.18%/0.3%	0.00%/0.4%
Sodium tripolyphosphate	0.70	0.53	0.35	0.18	0.00
Iota carrageenan	0.00	0.10	0.20	0.30	0.40
Additives *	6.45	6.45	6.45	6.45	6.45
Water	12.85	12.92	13.00	13.07	13.15
Brine	20.00	20.00	20.00	20.00	20.00
Meat	80.00	80.00	80.00	80.00	80.00
Total	100.00	100.00	100.00	100.00	100.00

* Salt (1.8%), curing salt (0.2%), sodium erythorbate (0.05%), ginger (0.2%), garlic (0.2%), starch (4%).

**Table 2:** Definitions of sensory characteristics for descriptive sensory analysis of five ostrich ham treatments.

Characteristics	Definition	Scale
Meaty aroma	The intensity of an overall meaty aroma, perceived by sniffing	0 = None; 100 = Strong
Ostrich meat aroma	The intensity of an ostrich meat aroma, perceived by sniffing	0 = None; 100 = Strong
Spicy aroma	The intensity of a spicy aroma, derived from ginger and garlic content, perceived by sniffing	0 = None; 100 = Strong
Meaty flavor	The intensity of an overall meaty flavor, perceived by tasting	0 = None; 100 = Strong
Ostrich meat flavor	The intensity of an ostrich meat flavor, perceived by tasting	0 = None; 100 = Strong
Spicy flavor	The intensity of a spicy flavor, derived from ginger and garlic content, perceived by tasting	0 = None; 100 = Strong
Mealiness	The degree of mealiness in the mouth, indicative of cohesiveness of sample, perceived by tasting	0 = None; 100 = Prominent

**Table 3:** Means (±SD) of the chemical and physical characteristics of five ostrich hams manufactured with decreasing Sodium tripolyphosphate levels (n = 4 per treatment) *.

	Sodium Tripolyphosphate/Iota Carrageenan Levels					LSD
	0.70%/0.0%	0.53%/0.1%	0.35%/0.2%	0.18%/0.3%	0.00%/0.4%	
Chemical composition						
Moisture (%)	73.2 ^b^ ± 0.0	73.4 ^b^ ± 0.1	73.8 ^ab^ ± 0.1	74.3 ^a^ ± 0.6	73.4 ^b^ ± 0.0	0.78
Fat (%)	2.9 ^a^ ± 0.1	2.8 ^a^ ± 0.3	2.5 ^a^ ± 0.2	2.8 ^a^ ± 0.3	2.7 ^a^ ± 0.2	0.61
Protein (%)	19.4 ^a^ ± 0.3	19.6 ^a^ ± 0.4	19.4 ^a^ ± 0.0	18.9 ^a^ ± 0.8	19.6 ^a^ ± 0.1	1.07
Ash (%)	4.0 ^a^ ± 0.0	3.7 ^ab^ ± 0.0	3.4 ^bc^ ± 0.3	3.3 ^bc^ ± 0.1	3.2 ^c^ ± 0.1	0.42
Phosphorus (%)	1.42	1.03	0.78	0.76	0.51	n/a
TME (calculated)^¤^	97.00	96.79	95.87	93.28	96.78	n/a
Product pH	6.24	6.23	6.26	6.21	6.20	n/a
Cooked yield (%)	86.0 ^d^ ± 0.9	88.1 ^c^ ± 0.2	91.9 ^b^ ± 2.4	94.1 ^a^ ± 1.5	92.5 ^ab^ ± 1.2	2.0
Instrumental color						
Lightness (L*)	48.1 ^c^ ± 1.9	49.4 ^bc^ ± 2.3	51.7 ^a^ ± 1.2	48.6 ^c^ ± 1.5	50.8 ^ab^ ± 2.2	1.53
Redness (a*)	9.8 ^a^ ± 0.6	9.1 ^b^ ± 0.7	8.3 ^c^ ± 0.5	9.5 ^ab^ ± 0.8	9.5 ^ab^ ± 0.9	0.59
Yellowness (b*)	11.4 ^b^ ± 0.5	12.4 ^a^ ± 1.2	12.7 ^a^ ± 1.2	12.6 ^a^ ± 0.9	13.0 ^a^ ± 0.7	0.77
Instrumental textural properties						
Hardness (N)	18.9 ^c^ ± 4.2	21.2 ^c^ ± 2.3	29.5 ^b^ ± 5.1	30.8 ^b^ ± 4.2	35.1 ^a^ ± 3.3	3.55
Cohesiveness (ratio)	0.42 ^c^ ± 0.64	0.44 ^bc^ ± 0.05	0.46 ^abc^ ± 0.03	0.49 ^ab^ ± 0.07	0.49 ^a^ ± 0.07	0.05
Gumminess (N)	8.3 ^c^ ± 2.0	10.9 ^bc^ ± 2.5	11.6 ^bc^ ± 6.5	14.3 ^ab^ ± 4.1	15.5 ^a^ ± 3.6	3.64
Springiness (mm)	5.3 ^c^ ± 0.6	5.1 ^c^ ± 0.5	5.6 ^bc^ ± 0.5	6.5 ^a^ ± 0.6	5.9 ^b^ ± 0.6	0.52

* Statistical analyses were performed on all data except for phosphorus, TME (Total Meat Equivalent: % Lean Meat Equivalent + % Total Fat) and pH, as these were measured/calculated only once per treatment; SD, Standard Deviation; LSD, Least Significant Difference (*p* = 0.05); ^a–d^ Means within the same row with different superscripts differ significantly (*p* ≤ 0.05), where L* represents white (100) to black (0), a* represents green (−ve values) to red (+ve values) and b* represents blue (−ve values) to yellow (+ve values).

**Table 4:** Means (±SD) for the sensory characteristics and hedonic scores (±SE) of five ostrich hams manufactured with decreasing Sodium tripolyphosphate levels (n = 4 per treatment).

	Sodium Tripolyphosphate/Iota Carrageenan Levels					LSD
	0.70%/0.0%	0.53%/0.1%	0.35%/0.2%	0.18%/0.3%	0.00%/0.4%	
Sensory characteristics						
Meaty aroma	25.4 ^ab^ ± 12.5	23.0 ^b^ ± 10.3	30.9 ^a^ ± 15.8	25.7 ^ab^ ± 14.3	23.6 ^b^ ± 15.3	5.88
Ostrich meat aroma	2.9 ^b^ ± 6.6	4.2 ^b^ ± 7.8	4.6 ^b^ ± 7.4	14.5 ^a^ ± 13.0	16.0 ^a^ ± 14.4	4.41
Spicy aroma	18.1 ^ab^ ± 17.6	19.2 ^a^ ± 16.8	13.1 ^b^ ± 11.8	4.6 ^c^ ± 8.2	6.3 ^c^ ± 10.7	5.48
Meaty flavor	26.8 ^b^ ± 14.4	25.5 ^b^ ± 14.5	40.2 ^a^ ± 18.5	22.1 ^b^ ± 15.4	22.2 ^b^ ± 16.4	5.08
Ostrich meat flavor	3.4 ^b^ ± 8.7	4.8 ^b^ ± 7.9	2.3 ^b^ ± 5.0	14.0 ^a^ ± 14.9	16.2 ^a^ ± 18.1	4.29
Spicy flavor	18.2 ^a^ ± 14.8	19.1 ^a^ ± 13.2	10.8 ^b^ ± 9.9	3.8 ^c^ ± 7.5	6.6 ^bc^ ± 11.0	5.38
Mealiness	17.5 ^a^ ± 14.4	18.8 ^a^ ± 16.2	11.8 ^b^ ± 10.0	3.5 ^c^ ± 4.2	5.7 ^c^ ± 8.8	4.29
Consumer preference						
Degree of liking	6.5 ^a^ ± 1.4	NE	6.4 ^a^ ± 1.4	NE	5.4 ^b^ ± 1.4	0.40

^a–c^ Means within the same row with different superscripts differ significantly (*p* ≤ 0.05); SD, Standard Deviation; SE, Standard Error; LSD, Least Significant Difference (*p* = 0.05); NE, Not Evaluated. Sensory characteristics were scored on 100-point scales, whereas the 9-point hedonic scale was used to score consumer preference.
